# Memory Alone Does Not Account for the Way Rats Learn a Simple Spatial Alternation Task

**DOI:** 10.1523/JNEUROSCI.0972-20.2020

**Published:** 2020-09-16

**Authors:** David B. Kastner, Anna K. Gillespie, Peter Dayan, Loren M. Frank

**Affiliations:** ^1^Department of Psychiatry and Behavioral Sciences, University of California, San Francisco, California 94143; ^2^Kavli Institute for Fundamental Neuroscience and Department of Physiology, University of California, San Francisco, California 94158; ^3^Max Planck Institute for Biological Cybernetics, Tübingen 72076, Germany; ^4^University of Tübingen, Tübingen 72074, Germany; ^5^Howard Hughes Medical Institute, San Francisco, California 94158

**Keywords:** behavioral modeling, learning and memory, reinforcement learning, rodent behavior

## Abstract

Animal behavior provides context for understanding disease models and physiology. However, that behavior is often characterized subjectively, creating opportunity for misinterpretation and misunderstanding. For example, spatial alternation tasks are treated as paradigmatic tools for examining memory; however, that link is actually an assumption. To test this assumption, we simulated a reinforcement learning (RL) agent equipped with a perfect memory process. We found that it learns a simple spatial alternation task more slowly and makes different errors than a group of male rats, illustrating that memory alone may not be sufficient to capture the behavior. We demonstrate that incorporating spatial biases permits rapid learning and enables the model to fit rodent behavior accurately. Our results suggest that even simple spatial alternation behaviors reflect multiple cognitive processes that need to be taken into account when studying animal behavior.

**SIGNIFICANCE STATEMENT** Memory is a critical function for cognition whose impairment has significant clinical consequences. Experimental systems aimed at testing various sorts of memory are therefore also central. However, experimental designs to test memory are typically based on intuition about the underlying processes. We tested this using a popular behavioral paradigm: a spatial alternation task. Using behavioral modeling, we show that the straightforward intuition that these tasks just probe spatial memory fails to account for the speed at which rats learn or the types of errors they make. Only when memory-independent dynamic spatial preferences are added can the model learn like the rats. This highlights the importance of respecting the complexity of animal behavior to interpret neural function and validate disease models.

## Introduction

Determining the causal relationship between animal behavior and its governing neural activity is a fundamental goal of systems neuroscience and is critical for understanding how aberrant neural processing underlies neuropsychiatric disease. However, the way in which we interpret animal behavior often rests on unidimensional and qualitative explanations of the factors at play. There are, for example, at least implicit claims that the elevated plus maze studies anxiety (Pellow et al., 1985; Walf and Frye, 2007), the forced swim test studies depression (Porsolt et al., 1978; Slattery and Cryan, 2012), prepulse inhibition studies sensorimotor gating (Swerdlow et al., 2000; Valsamis and Schmid, 2011), the Morris water maze studies spatial memory (Morris, 1984; Vorhees and Williams, 2006), and spatial alternation tasks study working memory (Shoji et al., 2012). Although these different components of cognition are likely necessary for the different behaviors, it cannot be assumed that they are the sole components responsible for the way in which animals perform the tasks. Instead, the successful understanding of animal behavior requires clear and quantitatively convincing elucidation of the factors that influence movement and decisions.

Here, we focus on spatial alternation, a class of behaviors widely used for studying hippocampal (Frank et al., 2000; Karlsson and Frank, 2008; Carr et al., 2012; Jadhav et al., 2012; Fernández-Ruiz et al., 2019), striatal (Gengler et al., 2005; Moussa et al., 2011), and prefrontal function and physiology (Aggleton et al., 1995; Delatour and Gisquet-Verrier, 1996, 2001; Jadhav et al., 2016; Shin et al., 2019) and as a cognitive test for animal models of neuropsychiatric disease (Sigurdsson et al., 2010; Mukai et al., 2019). Spatial alternation tasks, including the Y-, T-, and W-mazes, require animals to alternate visits to left and right maze arms between visits to the central arm. As memory for the immediate past choice or action (i.e., the past arm visited) is required for the behavior, changes in alternation behavior or differences in behavior between groups are typically interpreted as solely reflecting changes in memory processing. Here we use behavioral modeling to suggest that this interpretation, while intuitive, is critically incomplete. We demonstrate that, even making very generous assumptions, a purely memory-based model produces learning that is too slow and inconsistent with the way rats learn the task.

## Materials and Methods

### 

#### Experimental design and statistical analyses

All experiments were conducted in accordance with University of California San Francisco Institutional Animal Care and Use Committee and National Institutes of Health guidelines. Rat datasets were collected from Long–Evans rats that were fed standard rat chow (LabDiet 5001). To increase motivation, rats were food restricted to ∼85% of their basal body weight and provided with sweetened evaporated milk as reward in the task.

Ten male rats were run on the behavior in two cohorts of five animals each. At the start of the behavior, the rats were three to four months old. The rats came from four litters.

The track was the same as previous reports of the behavior (Karlsson and Frank, 2008). The track was elevated off of the ground. The arms were 76 cm long with reward wells at the end of each arm. The distance from the first to the third arm was also 76 cm with the arms equally spaced. The reward wells emitted an infrared beam, which the rat broke on visiting the well. The rats had a total of 15 sessions to learn the task across 5 d. Each session was 15 min long, and the three sessions within 1 d were separated by ∼2 h. At the start of the session the rat was placed at the base of the middle arm, on the opposite side of the arm from the reward well, facing the well.

Rewards were delivered according to the following rules. If the rat or agent is at any arm other than arm 2, the way to get reward is by going to arm 2. Once the rat or agent is at arm 2, the way to get reward is by going to the least recently visited arm between arm 1 or 3, whether or not that arm was previously rewarded. The one exception is if the rat or agent visits arm 2 on the first visit of the session, then reward would be delivered at either arm 1 or 3.

Before starting the alternation behavior all rats ran on a linear track for 3 d, 5 min each day, getting rewards by alternating between reward wells at each end of the linear track. This pretraining was done to familiarize the rats with how to get reward from the reward wells as well as habituate them to being on an elevated track. This pretraining is also consistent with previous reports of the behavior.

To evaluate the similarity or difference between the model learning rate and the learning rate of the average animal behavior, exponential fits were performed on the data and model with 99th confidence interval. If the values for the data and model did not overlap within the confidence interval, then the *p* value was determined to be <0.01.

#### Reinforcement learning (RL) agents

Given that the spatial alternation task could be framed as a partially observable Markov decision process, we adapted the working memory model of Todd et al. (2009) as the basis for our RL agent. The models specify rules governing propensities m(a,s) that contain the preferences of the agent of choosing arm a when the state is s. The models differ according to the various terms whose weighted sum defines the propensity.

The state is defined as the combination of the current arm location of the agent and the immediately preceding arm location of the agent, $s_{t}=\{a_{t-1,}a_{t}\}$. This is a simplification from the Todd et al. (2009) model, whereby $a_{t-1}$ is always placed into the memory unit, effectively providing perfect memory by setting the gating parameter for the memory unit to always update the memory unit. The first component of m(a,s) for all models is b(a,s), which is a 13×3 matrix containing the transition contingencies to arm a from state s. The reason for the additional states beyond just the 9 (3×3) arms by previous arms is to include the beginning of the session in the possible locations to allow for the inclusion of the first arm visit of a session. In so doing that adds 3+1 additional states since the animals can have just started the task and can be located at any of the three arms having previously just started the task.

To provide the agents with additional spatial and transitional preferences, we added components to the arm transition propensities. The first is an arm preference, $b^{\text{i}}(a)$ that is independent of the current state of the animal. The second is a preference for visiting arms that neighbor in space the current arm, $b^{n}\chi (a=a_{t}\pm 1)$, where χ() is the characteristic function that takes the value 1 if its argument is true (and ignoring arms outside the range 1 … 3) and $b^{n_{1}}$ is the (plastic) weight for this component. The neighbor arm transition preference contains only a single value which applies to all arms, which reflects the preference to go to any neighboring arm. The neighbor transition preference was applied equally in both directions when possible (i.e., if the agent was at the end of the track the neighbor bias could only be applied to one direction).

To determine the probability of visiting each of the arms from a given state, the total propensity is passed through a softmax such that:

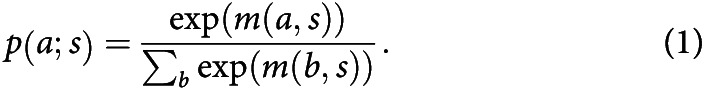


The agent's visit is then determined by a sample from this distribution. The choice of arm then determines the reward, r, which is either 0 or 1, based on the algorithm that governs the spatial alternation task. The probability of revisiting the current arm is set to zero, and the probabilities of going to the remaining arms sum to 1.

The model uses the REINFORCE policy gradient method (Williams, 1992) within the actor-critic framework of temporal difference learning, to update the propensities in the light of the presence or absence of reward. To do this, the agent maintains a state-long-run value approximation, V(s), which functions as a lookup table, with one component for each state. The reward determines the state-value prediction error:

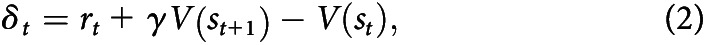
 where γ ϵ [0,1) is a parameter of the model called the temporal discounting factor, which determines the contribution of future rewards to the current state.

$\delta_{t}$ is then used to update the preferences all of the components of the propensities and V(s). The state-based transition component is updated according to the following rule:

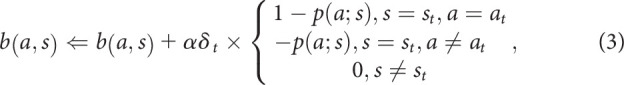
 where α ϵ [0,1] is a parameter of the model called the learning rate, which determines the amount by which all components of the propensities change based on the new information. The independent arm preference is updated according to the rule:

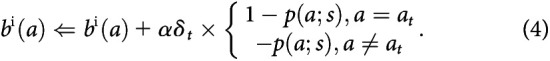


The strength of the neighbor arm preference is updated according to the rule:

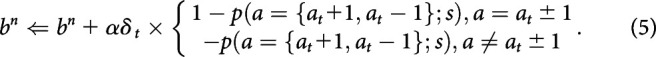


And, finally, the state-value approximation is updated according to the rule:

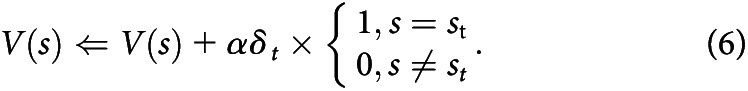


The learning rates, α, were the same for all of the updating rules. This does not need to be the case, but since we found that a single learning and forgetting rate fit the data well, we did not feel there was a need to increase the complexity of the models by increasing the number of parameters.

Initial conditions were set by adding a single value to the propensity to go to arm 1 and 3 across all states and for the enhanced memory models also for the independent arm bias for those arms. Given that the enhanced model had the extra independent arm terms, the single initializing value was lower for the enhanced model compared with the memory model. The initial value to go to arm 2 was initialized at 0. For the enhanced memory model, the transition bias was initialized at 0.

#### Model fitting

**Figure 1.**
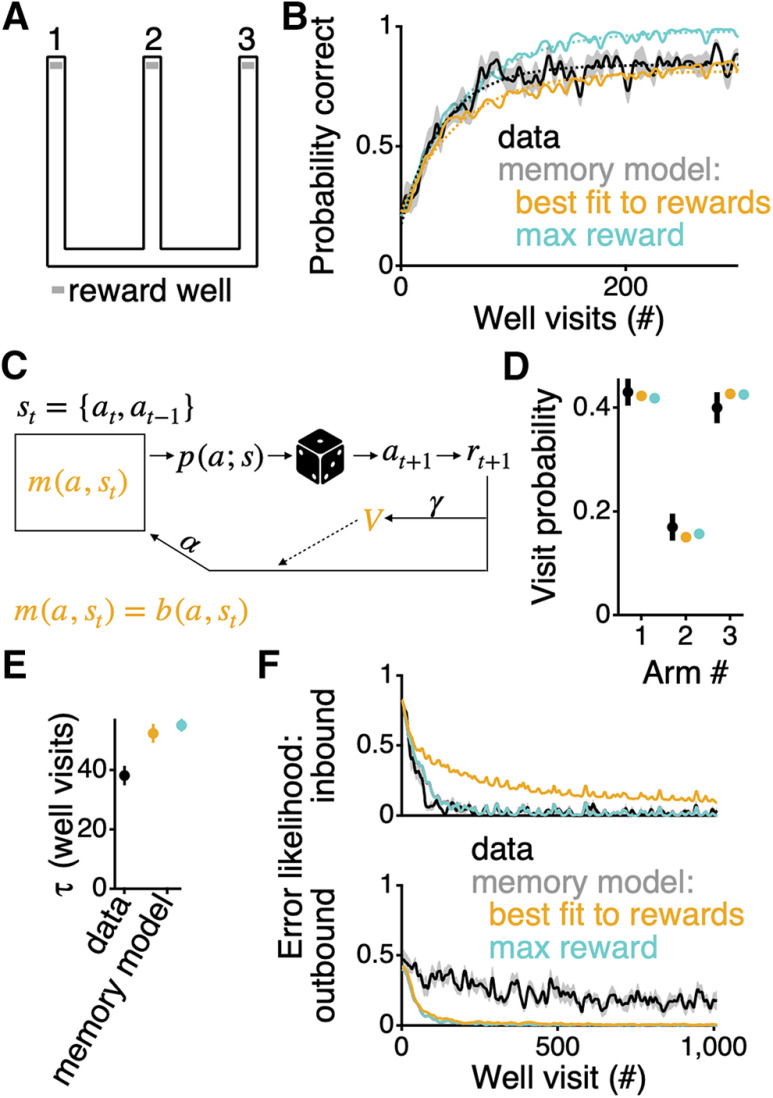
RL agent with memory does not learn a spatial alternation task in the same way as a group of rats. ***A***, Layout of the track. Reward wells were located at the end of the three arms of the track. ***B***, Probability of getting reward averaged across all rats (black; *n* = 10) and for the RL agent with just memory best fit (over 1012 trials) to the averaged data (orange) and fit to maximize reward (teal). The first 300 well visits are shown to highlight the trials over which the majority of the learning occurs. For each rat or single run of the agent, the presence or absence of reward over well visits was smoothed with a Gaussian filter with a SD of 2.25 well visits. For all curves, the width of the bar indicates SEM. Dotted lines show an exponential fit to the first 300 well visits. ***C***, Graphic of RL agent. Colored symbols, $m(a,s_{t})$ and V, reflecting the transition propensities and the value approximation, respectively, indicate the entities that change as the agent goes to arms, a, and does or does not get reward, r. The state of this agent, and therefore the probability of transitioning to each of the arms, p(a;s), is defined by the current arm location, $a_{t}$, and the previous arm location, $a_{t-1}$, of the agent. The propensities, $m(a,s_{t})$, are comprised only of the state-based transition matrix (i.e., the memory component). ***D***, Probability of visiting each of the arms within the first 10 trials averaged (±SEM) across all rats (black), across all repeats of the best fit model to the rewards (orange), and across all repeats of the model that maximizes the rewards (teal). ***E***, Values of τ for the exponential fits to the learning performance in panel ***B***. Vertical extent of the bars indicate the 99% confidence interval of the fit value. ***F***, Average inbound (top) and outbound (bottom) errors across all rats (±SEM; black), for the model that best fits the reward rate (orange), and for the fit that maximizes the reward (teal) as shown in panel ***B***. A third set of parameter values was fit to minimize the discrepancy between the inbound and outbound errors of the model and the averaged errors of the rats. These parameter values turn out to be very similar to those that maximize the total reward of the model, and the curves are therefore obscured by the teal lines (and so are not shown in part ***B*** or ***F***). Inbound and outbound errors for each animal were smoothed with a Gaussian filter with a SD of 2.25 errors and then interpolated to reflect well visits.

**Figure 2.**
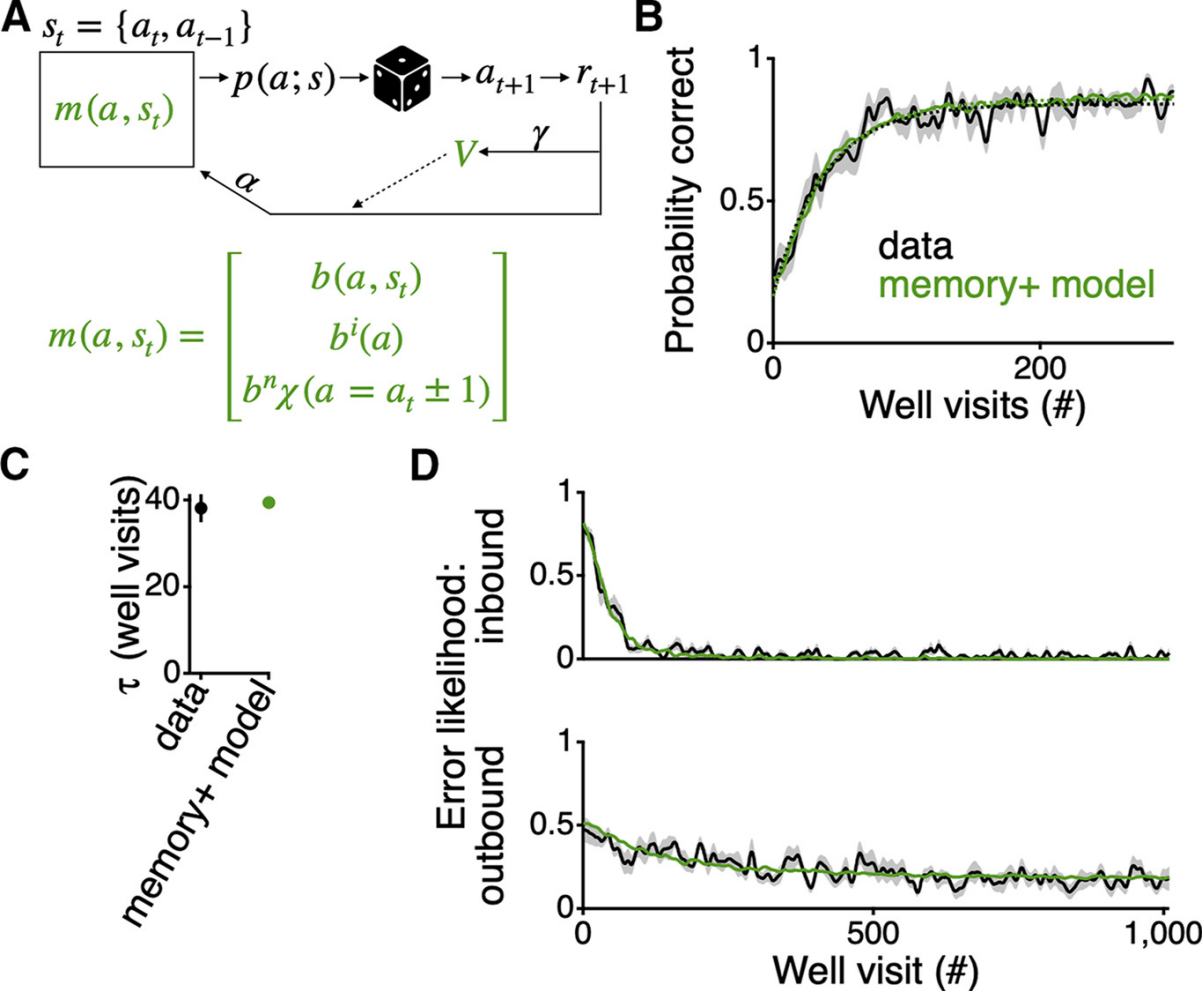
RL agent with memory and dynamic spatial preferences can learn a spatial alternation task as rapidly as a group of rats. ***A***, Graphic of RL agent. Colored symbols, $m(a,s_{t})$ and V, indicate the entities that change as the agent goes to arms, a, and does or does not get reward, r. The state of this agent, and therefore the probability of transitioning to each of the arms, p(a;s), is defined by the current arm location $a_{t}$ and the previous arm location $a_{t-1}$ of the agent. The propensities $m(a,s_{t})$ are comprised of the state-based transition matrix (i.e., the memory component) combined with an independent arm preference $b^{i}(a)$ and a neighbor transition preference $b^{n}\chi (a=a\pm 1)$. ***B***, Probability of getting reward averaged across all rats (black; *n* = 10) and for the RL agent with memory and the dynamic preferences for individual arms and neighbor transitions (green). The first 300 well visits are shown to highlight the time over which the majority of the learning occurs. For each rat or single run of the agent, the presence or absence of reward over well visits was smoothed with a Gaussian filter with a SD of 2.25 well visits. For all curves the width of the bar indicates SEM. Dotted lines show an exponential fit to the first 300 well visits. ***C***, Values of τ for the exponential fits to the learning performance in panel ***B***. Vertical extent of the bars indicate the 99% confidence interval of the fit value. ***D***, Average inbound (top) and outbound (bottom) errors across all rats (±SEM; black) and for the best fit model to those errors (green; different parameters than from ***B***). Inbound and outbound errors for each animal were smoothed with a Gaussian filter with a SD of 2.25 errors and then interpolated to reflect well visits.

**Figure 3.**
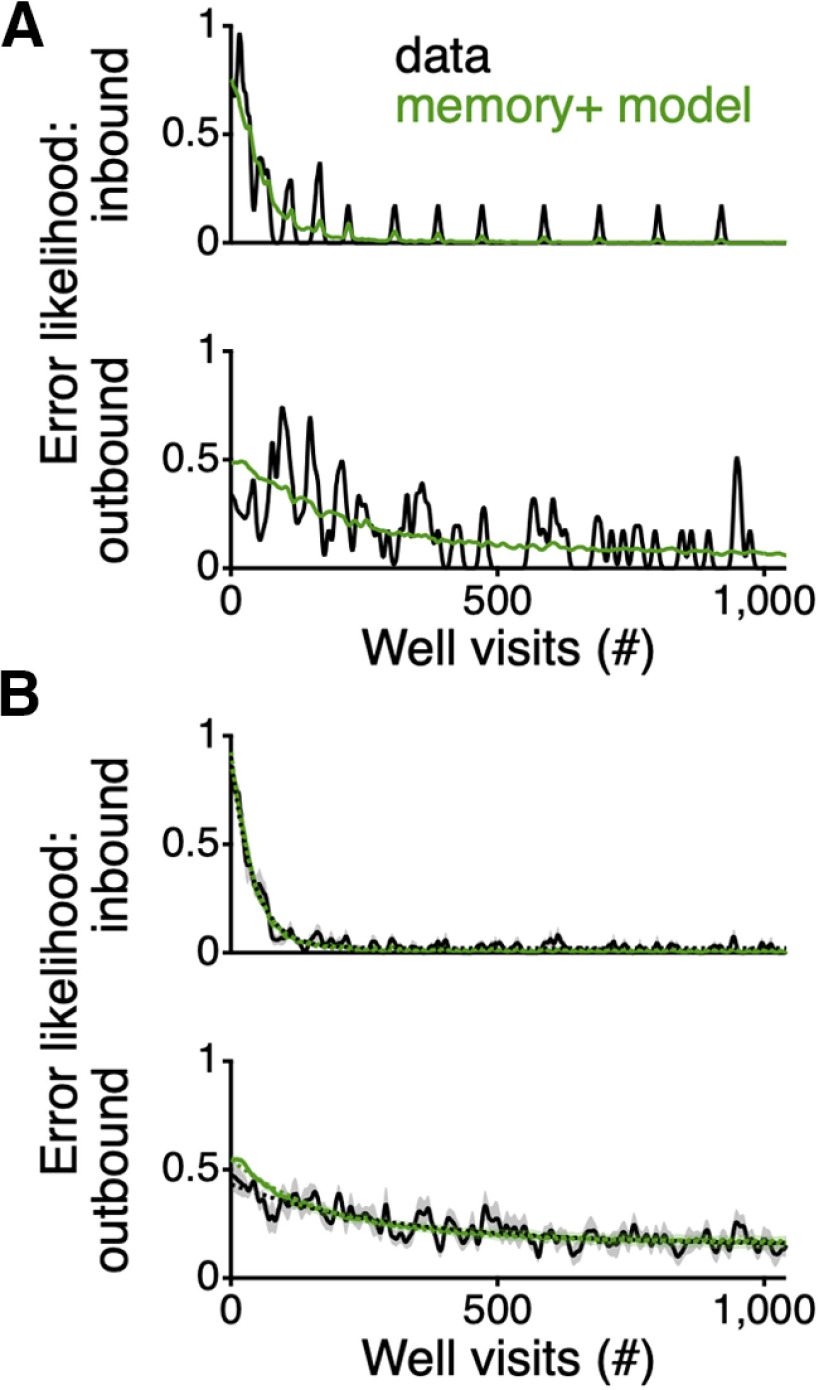
RL agent with memory and dynamic preferences fits spatial alternation learning behavior of individual rats. ***A***, Inbound (top) and outbound (bottom) error likelihood for an individual animal (black). Values smoothed with a Gaussian filter with a SD of 2.25 errors and then interpolated to reflect well visits. In green is the average behavior of 200 repeats of the model using the parameters that minimize the rms difference between the model and the animal, over all trials performed by that animal. The periodic bumps in the plot of the inbound errors reflect the beginning of a session where the rat or agent is likely to not start at arm 2 and thereby makes an inbound error. ***B***, Average inbound (top) and outbound (bottom) errors (±SEM) across all rats (black) and individual fits to each rat (green). Dotted lines show an exponential fit to the curves.

The agents were implemented in C++ and run and fit within Igor Pro (Wavemetrics). We fit the agents to average behavior of the rats and individual animals using the Approximate Bayesian Computation method (Lintusaari et al., 2017), as has previously been done for fitting rodent behavior with RL models (Luksys et al., 2009; Lloyd et al., 2012). For the fitting, we found the parameters that (1) minimized the average root mean square (rms) difference between the average performance of the rats and the average performance of 200 repeats of the agent (Fig. 1, “best fit to rewards”; see Fig. 2*B*); or (2) maximized the total rewards received by the model (Fig. 1, “max reward”); or (3) minimized the average rms difference between the inbound and outbound errors averaged across all animal and of the average of 200 different repeats of the model (see Fig. 2*D*); or (4) minimized the average rms difference between the inbound and outbound errors of the individual animal and of the average of 200 different repeats of the model (Fig. 3). The inbound and outbound fitting errors were summed with equal weighting to create the final fitting error. For all fitting categories, we used simulated annealing and ran the optimization at least four different times from different initial conditions. For all types of fitting to the average behavior of the animals, we fit to the first 1012 well visits; this was the maximum number of visits that all rats achieved. For the fitting to the individual animals, we fit to all of the well visits that that animal performed. For each run of the model we used the same random number generating seed to minimize the random fluctuations between parameter sets (Daw, 2011).

For the fit to the average behavior of the rats, the initial condition was set to match the initial reward rate of the model to the data. For the fits to the individual rats the initial condition was an additional fitting parameter and was therefore different for each rat.

#### Data and code availability

Code for the model as well as data for an example animal (animal from Fig. 3*A*) has been uploaded to a GitHub repository (https://github.com/dbkastner/threeArmWtrackModel.git). All data will made available on reasonable request.

## Results

We measured the performance of rats (*n* = 10) on a standard, three-arm, spatial alternation W-maze task (Fig. 1*A*,*B*). To gain reward, the rats had to learn, through trial and error, to alternate between visits to the outer arms after each visit to the center arm. A correct sequence of arm visits is, therefore, 2–3–2–1–2. To test the intuition that memory is solely responsible for the way animals learn the task, we adapted an RL agent with the capacity for working memory (Todd et al., 2009). This class of RL models has been used to learn common rodent behavioral tasks (Zilli and Hasselmo, 2008) and exhibits various features of rat behavior (Lloyd et al., 2012).

The RL agent chooses which arm to visit next based on its current state, s. The state is defined by two factors: the current arm location of the agent, $a_{t}$, and the previous arm visited by the agent, $a_{t-1}$, as maintained in a memory unit (Fig. 1*C*). In the original formulation by Todd et al. (2009), the agent had to learn whether to update or maintain the information in its memory unit. By contrast, we make the most generous possible assumption in favor of purely memory-based performance and always update the memory unit with the previously visited arm, thereby allowing the agent to have perfect memory for this task.

Each state has its own propensities, $m(a,s_{t})$, which determine the probabilities of making a transition to the other arms of the track (Fig. 1*C*). The propensities are updated at each trial through temporal difference learning within an actor-critic framework, such that, for example, for a given state if a given action led to reward when reward was not expected, its propensity is increased. This rule is a form of what is known as model-free (MF) RL (Sutton and Barto, 1998). There are two parameters that govern the performance of the agent, through changing the propensities: a learning rate α and a temporal discounting factor γ (for a full description of the modeling, see Materials and Methods).

To be able to have the potential to model the behavior of the rats accurately, we had to initialize the model to capture the initial biases expressed by the rats. In general, it is hard to measure the initial conditions of the rats independent of the task, since the rules for delivering reward are applied from the very first exposure to the environment. Nonetheless, we could approximate the initial conditions by measuring the probability of the rats visiting each of the arms within the first ten well visits. The rats show a strong initial preference for the two outer arms of the track (Fig. 1*D*), consistent with previous descriptions (Kim and Frank, 2009). Therefore, we set the initial propensities, $m(a,s_{t})$, of the model to match the initial error rate across the average of all rats (see Materials and Methods). In so doing, the model then matches the arm visit probabilities of the animals during the first ten well visits (Fig. 1*D*).

We found the model parameters that minimized the error between 200 repeats of the model and the average performance of the rats (Fig. 1*B*). Even with perfect memory, the model was unable to reach asymptotic performance as quickly as the rats. An exponential fit to the improvement in performance averaged across the rats had a trial number constant τ=38.1±3.3 trials (± 99% confidence interval), whereas the RL agent had τ=52.3±3.2 trials (Fig. 1*E*; p << 0.01). Thus, the RL agent learned the task ∼1.4 times slower than the rats.

If we maximized the rewards that the model could receive, instead of fitting to the average behavior of the animals, the model still had a learning rate slower than the rats (τ=55.1±2.1 trials; p << 0.01; Fig. 1*B*). However, these parameters provide a closer match to the initial learning trajectory. The major difference between the output of the model with the parameters that maximized the reward and the average reward of the animals is that the model had a higher asymptotic performance level. Thus, while this model could more closely replicate part of the behavior of the rats, it still failed to provide a complete account.

Developing a model that could provide a complete account of the behavior requires understanding not only the overall learning curve but also the specific errors made by the animals and the model. We therefore examined the patterns of errors across learning. In understanding how the animals learn this task, it has been helpful to consider the rules of the task (Kim and Frank, 2009; Jadhav et al., 2012; Fernández-Ruiz et al., 2019). These rules define two trial types, inbound and outbound. If the rat is at an outer arm, the way to get reward is to go to the center arm; we will refer to these trials as inbound trials. Any such trial on which the rat fails to go into the center arm is called an inbound error. Once at the center arm, then the only way the rat can get reward is to visit the less recently visited outer arm (i.e., if before going to the center arm 2 the rat came from arm 1, then it would have to go to arm 3 next to get a reward). We will refer to these trials as outbound trials and the corresponding error as an outbound error. In the traditional way of understanding this task, inbound trials do not require working memory whereas outbound trials do (Shin et al., 2019).

The rats learn the inbound rule much faster and more completely than the outbound rule (Fig. 1*F*), consistent with these rules being differentially sensitive to hippocampal manipulations (Kim and Frank, 2009; Jadhav et al., 2012; Fernández-Ruiz et al., 2019). The model fails to capture this difference. When we measure the inbound and outbound error rates of the model fit to the average behavior of the animals, the model distributes its errors very differently from the rats (Fig. 1*F*). Furthermore, this cannot be fixed by fitting the model directly to the inbound and outbound errors of the animals. Indeed, when we fit the memory model to minimize the difference between its error rates and the average inbound and outbound errors of the rats, the model more closely matches the inbound errors of the rats but deviates substantially from the trajectory of outbound errors of the rats (Fig. 1*F*). In the case of both fits, the model learns to perform the inbound trials more slowly than the outbound trials. This is exactly the opposite of what the rats do. These results provide evidence against the intuition that memory alone governs the way in which rats learn this task.

If memory is not the only computation responsible for learning this task, what else might be involved? We can modify the RL agent to formalize potential hypotheses about other contributions to the rapid learning of the rats. Two such assumptions are that (1) animals, in general, do not randomly visit locations and instead form preferences for certain locations over others; and (2) animals, in general, do not randomly transition between locations, but rather develop preferences for transitioning to neighbor locations.

We incorporated both of these dynamic preferences into the RL agent by adding additional contributions to the propensities whose strengths were initialized in a simple manner (see Materials and Methods) and were also updated through learning. These dynamic preferences combine with the memory component of the model to determine the choices of the agent (Fig. 2*A*). This enhanced model is now able to learn as rapidly as the rats, fitting well the average performance of the rats and closely mimicking the learning rate (τ=39.5±1.2 trials; non-significant difference as compared with the average performance of the rats; Fig. 2*B*,*C*). This enhanced model can also be fit to the average inbound and outbound errors of the rats (Fig. 2*D*). Furthermore, the parameters that were found when fitting the model to the average reward rate (α=0.120;γ=0.997) were very similar to those found when fitting to the error rates (α=0.133;γ=0.979). This indicates that the agent fit to the overall rewards of the rats makes similar types of errors as the rats, with reference to inbound and outbound errors.

The goal of enhancing the model with the dynamic preferences was to generate a hypothesis as to the additional computations that might underlie this simple spatial alternation task. We therefore performed subsequent analyses to understand the relative contributions of the two biases in enabling more rapid learning. We found that a model that just adds the independent arm preference to the memory learns more quickly than the original model but still does not learn as rapidly as the rats (τ=45.9±1.0 trials; p << 0.01 vs the rats). Additionally, a model that just adds the neighbor transition preference to the memory also learns faster than memory alone but still does not match the learning rate as well as the full model (τ=43.6±1.38 trials; p << 0.01). These results suggest that the two computations interact synergistically to enhance the learning.

The enhanced agent with all three components, memory, independent arm preference, and neighbor transition preference, not only can match the average behavior across all rats, it can also fit the way in which individual rats learn the task. We fit the enhanced model to all individual animals by minimizing the difference between the inbound and outbound error likelihood of the rats and 200 repeats of the agent. For the fits to the individual rats we added a third parameter to reflect the initial conditions (see Materials and Methods). The enhanced agent well captured the inbound and outbound errors of the rats (Fig. 3*A*,*B*), matching the different time courses to learn the inbound and outbound trials as well as the different asymptotic levels of these two error types. That match was reflected in more similar values of the learning rates: the model τ=41.6±0.4 for the inbound errors overlapped with τ=41.1±1.3 for the inbound errors for the average rat behavior (p > 0.05 vs the rats). The model τ=183.3±3.1 for the outbound errors reflects slower learning than for the inbound trials, although that value does reflect faster changes than the τ=306.8±41.5 for the outbound errors for the average rat behavior (p << 0.01). A subsequent analysis revealed that the differences in τ between the enhanced model and the animals could be explained largely by differences in the offset and scale of the exponential fits, as constraining those parameters resulted in very similar decay values. Thus, this model recapitulates the different learning rates for the inbound and outbound components of the task; however, the rats still show a slight deviation from the model in their initial outbound error rate, indicating further potential components beyond memory that might still underlie the behavior of the animals.

## Discussion

Here, we have shown that a core, and largely unquestioned, assumption underlying the interpretation of spatial alternation behavior is likely incorrect. Memory alone, as implemented in a MF RL system, does not account for the way in which rats learn even a simple spatial alternation task on a W-maze (Fig. 1). To better understand the computations that underlie a behavior, traditionally, many animals would be run on various, but conceptually related tasks, using lesions of various brain regions (Delatour and Gisquet-Verrier, 1996, 2001; Ennaceur et al., 1997; Gisquet-Verrier and Delatour, 2006). Here, we have taken a more direct approach using precisely defined models. To generate a hypothesis as to what might account for the rapidity of the learning, we posit that the task also draws on dynamic preferences to visit and transition between neighboring arms. A model that incorporates such biases can learn as quickly as the rats (Fig. 2) and can well fit the behavior of individual animals (Fig. 3).

It is important to clarify what these results mean. We have not proven that it is impossible that a process that only depends on memory can learn this task as rapidly as the rats. It remains possible (but we argue unlikely) that a different, purely memory-based model might be able to replicate the behavior. However, short of a quantitative demonstration of this, we suggest that our results shift the burden of proof, making it inappropriate to posit that memory is solely responsible for the behavior.

Dynamic preferences provide a simple explanation for what else might be involved in the rapid learning of the rats. However, other computations, as implemented in different processes, could underlie the rapid learning. For instance, some form of model-based RL (Gershman and Niv, 2010; Lake et al., 2017) could be designed to model the behavior, but such a formulation would also require adding putative cognitive biases or schema that go beyond simple memory. Furthermore, it would likely require some modification to cause the model-based agent to slow down its learning to match the quality of fit to the animal behavior that we exhibited. Future experiments will be necessary to determine the actual additional computations involved in this behavior; and, given model mimicry, neural data might also have to be called on.

Animal behavior is complex. To make progress in understanding the causal relationship between neural activity and behavior, it is critical to respect and account for that complexity. Our results demonstrate that richer accounts are necessary even to encompass apparently simple behaviors and illustrate the benefits and necessity of moving toward quantitative models of behavior.
